# *Haemophilus ducreyi* Associated with Skin Ulcers among Children, Solomon Islands

**DOI:** 10.3201/eid2010.140573

**Published:** 2014-10

**Authors:** Michael Marks, Kai-Hua Chi, Ventis Vahi, Allan Pillay, Oliver Sokana, Alex Pavluck, David C. Mabey, Cheng Y. Chen, Anthony W. Solomon

**Affiliations:** London School of Hygiene & Tropical Medicine, London, UK (M. Marks, D.C. Mabey, A.W. Solomon);; Hospital for Tropical Diseases, London (M. Marks, D.C. Mabey; A.W. Solomon);; Centers for Disease Control and Prevention, Atlanta, Georgia, USA (K.-H. Chi, A. Pillay, C.Y. Chen);; Ministry of Health and Medical Services, Honiara, Solomon Islands (V. Vahi, O. Sokana);; Task Force for Global Health, Atlanta (A. Pavluck)

**Keywords:** *Haemophilus ducreyi*, chancre, chancroid, yaws, ulcer, Buruli ulcer, skin, lesion, cutaneous, nongenital, bacterial, ulcerative, pediatric, children, Solomon Islands

## Abstract

During a survey of yaws prevalence in the Solomon Islands, we collected samples from skin ulcers of 41 children. Using PCR, we identified *Haemophilus ducreyi* infection in 13 (32%) children. PCR-positive and PCR-negative ulcers were phenotypically indistinguishable. Emergence of *H. ducreyi* as a cause of nongenital ulcers may affect the World Health Organization’s yaws eradication program.

Bacterial ulcerative skin diseases are a common cause of illness in the developing world (*1*). Some of these diseases, including Buruli ulcer, caused by *Mycobacterium ulcerans*, and yaws, caused by *Treponema pallidum* subspecies *pertenue*, occur only in tropical and subtropical climates. Yaws is endemic in the Solomon Islands, where ≈15,000 cases per year are reported (*2*). In 2012, the World Health Organization (WHO) launched a worldwide yaws eradication program based on treatment by mass distribution of azithromycin and monitoring for skin ulcers (*3*).

Reports suggest that *Haemophilus ducreyi*, the causative organism of chancroid, a sexually transmitted infection, may be associated with nonsexual transmission of nongenital ulcers of the skin in persons from the Pacific region (*4*,*5*). If this organism is a common cause of skin ulcers in the region, this factor has crucial implications for the yaws eradication strategy. PCR has been shown to be highly sensitive and specific for diagnosing chancroid (*6*). We used real-time PCR to detect *H. ducreyi* in skin ulcer samples collected during a survey for yaws in the Solomon Islands.

## The Study

We conducted a cross-sectional survey for yaws in the Western Province and Choiseul Province of the Solomon Islands in 2013. In each province, we chose 25 clusters using a probability-proportionate-to-size method. In each cluster, we selected 30 houses by random sampling; children 5 to 14 years of age living in those houses were invited to participate. Informed written consent was obtained from the children’s parents. 

Children underwent standardized examination. We recorded location, classification, and duration of skin lesions and yaws treatment history using the LINKS system (*7*, http://www.linkssystem.org/). Lesions were classified by using the WHO pictorial grading scheme for yaws (*8*). Tenderness was classified based on reports by children. Blood samples were collected from all children. For children with exudative skin lesions, a sample for PCR was collected by rolling a sterile cotton-tipped swab across the lesion and placing it in a cryotube pre-filled with 1.2 mL of AssayAssure solution (Thermo Fisher Scientific, Waltham, MA, USA). Samples were transferred to Honiara National Referral Hospital within 5 days and stored at −20°C. Serum samples were placed on dry ice and shipped to the London School of Hygiene & Tropical Medicine and lesion samples to the US Centers for Disease Control and Prevention.

Serum samples were tested by using *T. pallidum* particle agglutination (Mast Diagnostics, Merseyside, UK) at the London School of Hygiene & Tropical Medicine. For samples with a positive *T. pallidum* particle agglutination, a rapid plasma regain test was performed (Deben Diagnostics, Ipswich, UK). DNA was extracted from lesion samples in a CDC laboratory by using iPrep PureLink gDNA blood kits and the iPrep purification instrument (Life Technologies, Grand Island, NY, USA). A real-time duplex PCR targeting the DNA polymerase I gene (*polA*, *tp0105*) of pathogenic treponemes (which detects all 3 *T. pallidum* subspecies) and the human *RNase P* gene (to monitor for PCR inhibition) was performed by using a Rotor-Gene-Q real-time PCR instrument (QIAGEN Inc., Valencia, CA, USA) (*9*). Negative (no-template) control and positive controls for *T. pallidum* DNA were included in each PCR run. Considering reports of *H. ducreyi* and the occurrence of *M. ulcerans* in Papua New Guinea, immediately north of the Solomon Islands, we performed a second duplex real-time PCR for *M*. *ulcerans* and *H. ducreyi* on all samples by using previously validated targets (*10*,*11*).

For the purpose of analysis, lesions were classified as acute (<4 weeks) or chronic (>4 weeks). A rapid plasma regain titer ≥1/4 was considered positive. Fisher exact test was used to compare characteristics of patients whose lesions contained *H. ducreyi* with patients whose lesions did not contain *H*. *ducreyi*. Analyses were performed by using STATA 13.1 (http://www.stata.com/).

During the survey, 1,497 children were examined. Samples for PCR were collected from 41 children who had exudative lesions (19 male, median age 8 years). Twenty-two children had ulcerative lesions from which a sample could not be collected. Twelve (29.3%) children had positive results for yaws from serologic testing, but no DNA evidence of *T. pallidum* subsp. *pertenue* or *M. ulcerans*, causative organisms of yaws and Buruli ulcer, respectively, was detected in any sample. *H. ducreyi* DNA was amplified from 13 (32%) samples (Figure). PCR inhibitors were not found in any samples. Clinical data were incomplete for 2 participants. There were no notable differences in the recorded characteristics of skin lesions or in the serologic status of patients in whose ulcers *H ducreyi* DNA was found compared with those in which *H ducreyi* was not found (Table).

**Figure:**
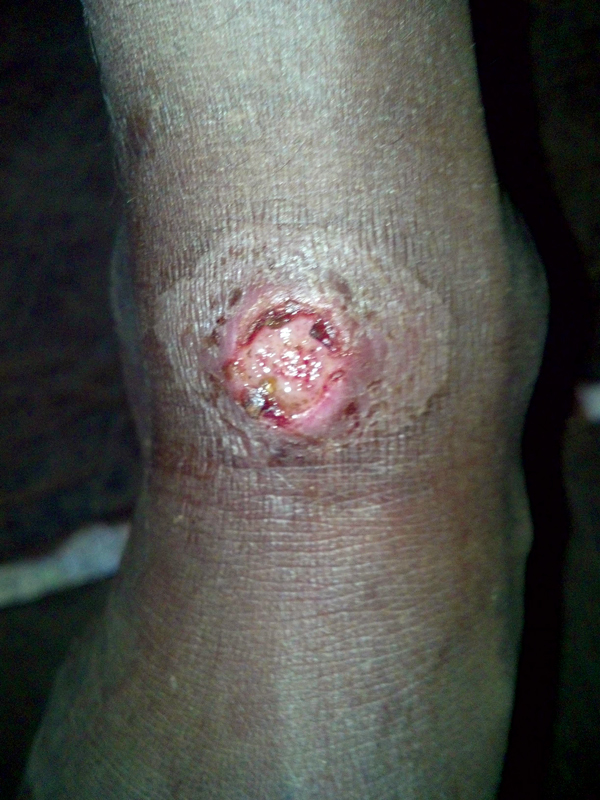
Example of lesion from which sample was obtained and *Haemophilus ducreyi* DNA was amplified, Solomon Islands, 2013. Photograph ©2014 Michael Marks.

**Table:** Comparison of skin ulcer samples from 41 patients tested for *Haemophilus ducreyi*, Solomon Islands, 2013*

Characteristic	No. (%) samples tested for *H. ducreyi* by real-time-PCR, 95% CI		p value
	Positive, n = 13	Negative, n = 28	
Male sex	8 (62), 32%–86%	10 (36), 19%–56%	0.179
Location of lesion on leg	12 (92), 64%–99%	21 (96), 80%–99%	0.561
Duration <4 weeks	7 (54), 25%–81%	14 (54), 33%–73%	0.632
Painful lesion	8 (62), 32%–86%	17 (65), 44%–83%	0.542
Sample TPPA-positive	6 (46), 19%–75%	17 (61), 41%–78%	0.503
Sample TPPA- and RPR-positive	5 (38), 14%–68%	7 (25), 11%–45%	0.469

*TPPA, *Treponema pallidum* particle agglutination; RPR, rapid plasma regain test.

## Conclusions

*H. ducreyi* is frequently present in skin ulcers of children in the Solomon Islands, and lesions containing *H ducreyi* DNA were similar in location, duration, and tenderness to lesions in which *H ducreyi* was not found. Papua New Guinea reported a similar finding (*12*). Experimental models of chancroid have demonstrated that injection of *H. ducreyi* into the epidermis and dermis causes nongenital skin disease, suggesting that *H. ducreyi* may be the cause of some ulcers in our survey (*13*). Similar to results for experimental models, *H. ducreyi* DNA was found more frequently in samples collected from boys (8/13; p = 0.179), although this difference was not statistically significant. It is possible that the difficulty of collecting samples for molecular testing, the lack of facilities to enable collection of samples for culture in affected areas, and the precise culture requirements of *H. ducreyi* have notably delayed recognition of this association (*14*).

Lesions associated with *H*. *ducreyi* were found in patients with positive and negative serologic test results for *T. pallidum* subsp. *pertenue*. It is likely that patients with positive serologic test results represent latent yaws with an alternative etiologic agent causing the current lesion. The possibility that there are alternative causes of childhood skin ulcers in the Pacific region could have implications for WHO’s yaws eradication strategy, which is based on detection of suspected clinical cases. Although azithromycin is effective in treating genital strains of *H. ducreyi* and experimental nongenital lesions (*15*), further studies are needed to confirm efficacy in nongenital lesions in a clinical setting. The emerging data suggest that surveillance strategies should routinely require molecular diagnostics. 

A causative agent was not identified in a large proportion of lesion samples. A variety of possible reasons exist for this, including the fact that some lesions were noninfectious, such as insect bites, some numbers of organisms were below current limits of detection of real-time-PCR, or that other organisms, such as staphylococci, for which PCR was not performed, caused these lesions. The sample collection/transport media and PCR assays we used varied from those used by Mitjà et al (*12*), but it is unclear to what extent this effected our results. A single sample was collected per patient, but several patients (n = 8, 19.5%) had >1 skin lesion. Swabbing every lesion may have increased the diagnostic yield for *H. ducreyi* and/or *T. pallidum* subsp. *pertenue*. Further studies to explore causes of skin ulcers in this community are needed to better inform disease control efforts.

Because it was not anticipated that *H*. *ducreyi* DNA would be found in nongenital skin lesions, we did not prospectively collect data on regional lymphadenopathy; however, we did not notice marked lymphadenopathy or bubo formation. Collection of samples for culture and sequencing of the *H*. *ducreyi* genome are needed to inform our understanding of relatedness of these strains to genital strains.

This study has 2 main limitations. First, the number of samples tested was small. Second, lesion samples were tested for only 3 organisms, raising the possibility that other organisms caused a large proportion of skin ulcers. Despite these limitations, this study clearly demonstrates that *H*. *ducreyi* is frequently present in childhood skin ulcers in this yaws-endemic community. Further studies of the epidemiology, microbiology, and response to treatment for this newly described pathogen–disease association are required.
